# Trehalose promotes atherosclerosis regression in female mice

**DOI:** 10.3389/fcvm.2024.1298014

**Published:** 2024-02-16

**Authors:** Sabrina Robichaud, Valérie Rochon, Christina Emerton, Thomas Laval, Mireille Ouimet

**Affiliations:** ^1^ Biochemistry, Microbiology and Immunology, Faculty of Medicine, University of Ottawa, Ottawa, ON, Canada; ^2^ Cardiovascular Metabolism and Cell Biology Laboratory, University of Ottawa Heart Institute, Ottawa, ON, Canada

**Keywords:** atherosclerosis, autophagy, regression, trehalose, cholesterol, inflammation

## Abstract

**Introduction:**

Atherosclerosis is a chronic inflammatory disease caused by the deposition of lipids within the artery wall. During atherogenesis, efficient autophagy is needed to facilitate efferocytosis and cholesterol efflux, limit inflammation and lipid droplet buildup, and eliminate defective mitochondria and protein aggregates. Central to the regulation of autophagy is the transcription factor EB (TFEB), which coordinates the expression of lysosomal biogenesis and autophagy genes. In recent years, trehalose has been shown to promote TFEB activation and protect against atherogenesis. Here, we sought to investigate the role of autophagy activation during atherosclerosis regression.

**Methods and results:**

Atherosclerosis was established in C57BL/6N mice by injecting AAV-PCSK9 and 16 weeks of Western diet feeding, followed by switching to a chow diet to induce atherosclerosis regression. During the regression period, mice were either injected with trehalose concomitant with trehalose supplementation in their drinking water or injected with saline for 6 weeks. Female mice receiving trehalose had reduced atherosclerosis burden, as evidenced by reduced plaque lipid content, macrophage numbers and IL-1β content in parallel with increased plaque collagen deposition, which was not observed in their male counterparts. In addition, trehalose-treated female mice had lower levels of circulating leukocytes, including inflammatory monocytes and CD4^+^ T cells. Lastly, we found that autophagy flux in male mice was basally higher than in female mice during atherosclerosis progression.

**Conclusions:**

Our data demonstrate a sex-specific effect of trehalose in atherosclerosis regression, whereby trehalose reduced lipid content, inflammation, and increased collagen content in female mice but not in male mice. Furthermore, we discovered inherent differences in the autophagy flux capacities between the sexes: female mice exhibited lower plaque autophagy than males, which rendered the female mice more responsive to atherosclerosis regression. Our work highlights the importance of understanding sex differences in atherosclerosis to personalize the development of future therapies to treat cardiovascular diseases.

## Introduction

Atherosclerosis is characterized by the deposition of lipid-laden cells, known as foam cells, within the intimal layer of medium and large arteries (1). During the progression of atherosclerosis, high levels of cholesterol-rich low-density lipoprotein (LDL) particles enter the intimal layer of the arteries, where they become modified and activate the overlying endothelium. In turn, activated endothelial cells secrete cytokines and chemokines that recruit circulating monocytes. Upon entry, monocytes differentiate into macrophages that engulf the modified LDL (2, 3). The excess intracellular cholesterol undergoes esterification for storage within lipid droplets to prevent cellular lipotoxicity, resulting in macrophage foam cell formation (4).

In recent years, several mouse studies have reported that atherosclerotic plaque growth is halted upon the reversal of hypercholesterolemia, and these plaques undergo remodelling. This process was coined atherosclerosis “*regression.”* A regressing atherosclerotic plaque exhibits one or more of the following: reduced size, reduced cholesterol content, dampened inflammation, reduced foam cell numbers, increased collagen content and/or increased fibrous cap size (5, 6). However, our understanding of the mechanisms that promote atherosclerosis regression is incomplete. Nonetheless, therapies that reduce circulating cholesterol levels in humans have provided clinical evidence that human atherosclerotic plaques can undergo regression, including remodelling and a reduction of overall plaque area (7, 8). Still, the amelioration of atherosclerosis in humans following hypercholesterolemia reversal is modest and more effective therapies to reverse this disease are needed. Here, we tested if autophagy activation promotes atherosclerosis regression.

Autophagy is a constitutive process that promotes cell homeostasis by degrading intracellular components to their constituent building blocks (i.e., amino acids, fatty acids) (9, 10). Autophagy can facilitate the removal of dysfunctional mitochondria (11), protein aggregates (12), inflammasomes (13) and lipid droplets (14, 15), all of which accumulate during atherogenesis (16, 17). We and others have shown that during atherosclerosis development, plaque foam cell autophagy becomes dysfunctional (12, 13, 18, 19). One possibility is that functional autophagy cannot ensue due to decreased lysosomal degradative capacity during atherosclerosis (20–22). Central to the regulation of autophagy and lysosome function is the transcription factor EB (TFEB) (23). Nuclear translocation of TFEB leads to the expression of genes involved in autophagosome and lysosome biogenesis, increasing processes such as autophagy, phagocytosis, lipid catabolism, exocytosis, and endocytosis (23–25). Furthermore, activation of TFEB using the disaccharide trehalose reduced atherosclerosis progression (26, 27), highlighting the therapeutic potential of autophagy activation for atherosclerosis treatment.

Human patients diagnosed with cardiovascular disease already have established atherosclerotic plaques and are typically prescribed a lipid-lowering therapy, such as statins. As such, herein, we sought to determine whether autophagy activation subsequent to cholesterol lowering could promote the regression of pre-existing atherosclerotic plaques in a pre-clinical model of atherosclerosis. First, we induced atherogenesis in C57BL/6N mice by overexpression of the gain-of-function mutant PCSK9 followed by 16 weeks of Western diet feeding. Following the development of atherosclerosis, atherosclerosis regression was initiated by a switch to a chow diet concurrent with the administration of trehalose for 6 weeks. We found that trehalose reduced plaque lipid content, inflammation, and increased collagen content in female mice but not in male mice. Interestingly, we observed lower basal autophagy in the plaques of female mice compared to males, which rendered the female mice more responsive to both the diet switch and trehalose treatment. Our data suggests that differential autophagy flux between sexes underlies their distinct capacities to respond to therapeutic interventions, highlighting the need for adapted therapies for better efficiency in the treatment of atherosclerosis.

## Materials and methods

### Animals

Male and female C57BL/6N (Charles River) mice 6 weeks of age were injected intraperitoneally with 1 × 10^11^ particles of AAV-PSCK9 (University of Pennsylvania), and 7 days post-injection, the mice were started on a Western Diet (Envigo, TD88137) for 16 weeks. Mice were randomized to the baseline, regression saline or regression trehalose groups. The baseline group was sacrificed following the 16 weeks of Western Diet. To induce atherosclerosis regression, the regression saline and trehalose groups were switched to a standard laboratory chow diet for 6 weeks, as previously described (5) to induce atherosclerosis regression. During the chow diet feeding, mice received bi-weekly subcutaneous injections of either saline or trehalose (Fisher, 2 g/kg). Mice receiving the trehalose injections received 3% trehalose water *ad libitum*. The animals were fasted for 4 h before sacrifice or blood collection. All animal procedures were approved by the University of Ottawa Animal Care and Use Committee.

### Plaque histology

Aortic sinuses were immediately frozen at sacrifice in OCT (Fisher) and stored at −80°C until ready to be sectioned. Embedded sinuses were cut at 10 µm of thickness, and serial sections were collected. Neutral lipid content was assessed using Oil Red O staining as previously described (19). Masson Trichrome (Sigma) staining was performed according to the the manufacturer's instructions. Atherosclerotic plaques were imaged using an Aperio Versa 8 (Leica) microscope at 20x (NA 0.8). Plaque area was quantified as previously described (28) using FIJI (NIH) and was blinded according to sex and treatment groups.

### Liver histology

Livers were fixed in 10% formalin at sacrifice and cryopreserved in 30% sucrose before embedding in OCT. Livers were cryosectioned at a thickness of 10 µm. Neutral lipid content was assessed using Oil Red O as above. Liver sections were imaged using an Aperio Versa 8 (Leica) microscope at 40x (NA 0.8). Neutral lipid quantification was performed using FIJI (NIH) and was blinded with respect to sex and treatment groups.

### Plasma cholesterol measurement

Blood was collected following a 4 h fast by saphenous vein bleed or cardiac puncture at the time of sacrifice in EDTA-coated microtubes (Sarstedt). Blood was centrifuged at 900xg for 10 min at 4°C, and the plasma was collected. Total plasma cholesterol was measured using the Infinity Cholesterol Liquid Stable Reagent (Thermo Scientific) as per the manufacturer's recommendation.

### Immune phenotyping

Whole blood was collected following 16 weeks of Western Diet feeding and at 5 weeks of chow diet feeding by saphenous vein bleeds. Blood was lysed for 5 min at room temperature using BD Pharmlyse (BD Biosciences). Leukocytes were blocked for 5 min at room temperature using FcBlock (BD Biosciences) in FACS buffer (3% FBS in PBS), followed by incubation with primary conjugated antibodies for 20 min on ice. The antibodies used are listed in Supplementary Table S1. Cells were incubated on ice with the viability marker Fixable Viability Stain 700 (BD Biosciences) for 30 min. Cells were fixed on ice with BD Cytofix (BD Biosciences) for 15 min. Samples were acquired using BD FACS Aria IIIu (BD Biosciences). Sample analysis was performed using FlowJo 10.8.2 (BD Biosciences).

### Immunofluorescence

Aortic sinuses were fixed in 4% paraformaldehyde (Fisher) for 10 min at room temperature and washed in PBS. The sections were blocked and permeabilized in 10% normal horse serum (Vector) and 0.1% Triton X-100 (Fisher) for 30 min at room temperature. Primary antibodies were diluted in 1% normal horse serum and incubated for 2 h at room temperature or overnight at 4°C. Secondary antibodies were incubated at room temperature for 1 h 30 min in 1% normal horse serum. Nuclei were stained using DAPI (Invitrogen) for 20 min at room temperature. Slides were mounted using Prolong Glass (Invitrogen) and #1.5 coverslips (Ibidi). The aortic sinuses were imaged using a Zeiss AxioObserver Z1 at 20x (NA 0.8). Images were quantified using FIJI (NIH) and blinded with respect to sex and treatment groups. The antibodies used are listed in Supplementary Table S1.

### Aortic digests and autophagy flux quantification

Thoracic aortas were surgically exciced and digested as previously described (19). Single cell suspensions were treated ex vivo for two hours with either DMSO (Fisher BioReagent) or Bafilomycin (Sigma) in DMEM (Gibco) supplemented with 10% FBS (Gibco). Cells were stained for 30 min with the CYTO-ID Green Detection Reagent from the CytoID Autophagy Detection Kit 2.0 (Enzo Life Sciences) and Lysotracker Red DND-99 (Invitrogen). The aortic digest was incubated with FcBlock (BD Biosciences) followed by incubation with primary conjugated antibodies for 30 min on ice. Cells were stained for neutral lipids using LipidTOX Deep Red (Invitrogen) (19). Samples were aquired using BD FACS Aria IIIu (BD Biosciences). Sample analysis was performed using FlowJo 10.8.2 (BD Biosciences).

### Cell culture

Peritoneal macrophages were harvested from 8-week old male and female C57BL/6N mice (Charles River) 3 days after intraperitoneal injection of 1 ml 3% thioglycolate (BD Difco), and cultured in DMEM media supplemented with 10% FBS and 1% penicillin-streptomycin (Gibco). Macrophages were lipid-loaded by incubation with 50 µg/ml of aggregated LDLs (agLDL) for 24 h, followed by an overnight equilibration in DMEM supplemented with 2 mg/ml of fatty acid-free BSA (Sigma), as previously (14, 15). For autophagy flux analyses, cells were treated with DMSO or Bafilomycin 2 h prior harvest.

### Western blotting

Peritoneal macrophages were lysed in 2X Laemmli Sample Buffer (Bio-Rad) containing β-mercaptoethanol and boiled at 95°C for 5 min. Samples were run on 8%–16% Criterion TGX Stain Free Pre-cast Gels (BioRad) and UV-activated for total protein quantification using the ChemiDoc XRS + System (Bio-Rad). Proteins were transferred onto 0.22*μ*m PVDF Membranes (Bio-Rad) using the Trans-Blot Turbo Transfer System (Bio-Rad). Immunoblotting for indicated proteins was performed with primary antibodies overnight at 4°C followed by horseradish peroxidase conjugated secondary antibodies for 1 h at room temperature. Proteins were developed using using either Clarity (Bio-Rad) or Clarity Max (Bio-Rad) ECL Substrates and imaged on the ChemiDoc XRS + system (Bio-Rad).

### Statistical analysis

The data is presented as mean ± SEM. Statistical analyses were performed using Prism Graphpad V 10.0.2 (Graphpad Software Inc). All samples were analyzed for outliers using the ROUT test. The normal distribution of the data points was assessed using the Shapiro-Wilk test. Statistical significance was determined using a 2-tailed Student *T* test, 1-way ANOVA or 2-way ANOVA using Holm-Sidak multiple comparison correction. A *P*-value of <0.05 was considered as statistically significant.

## Results

### The administration of trehalose did not alter body composition nor circulating cholesterol in female mice

**Figure 1:**
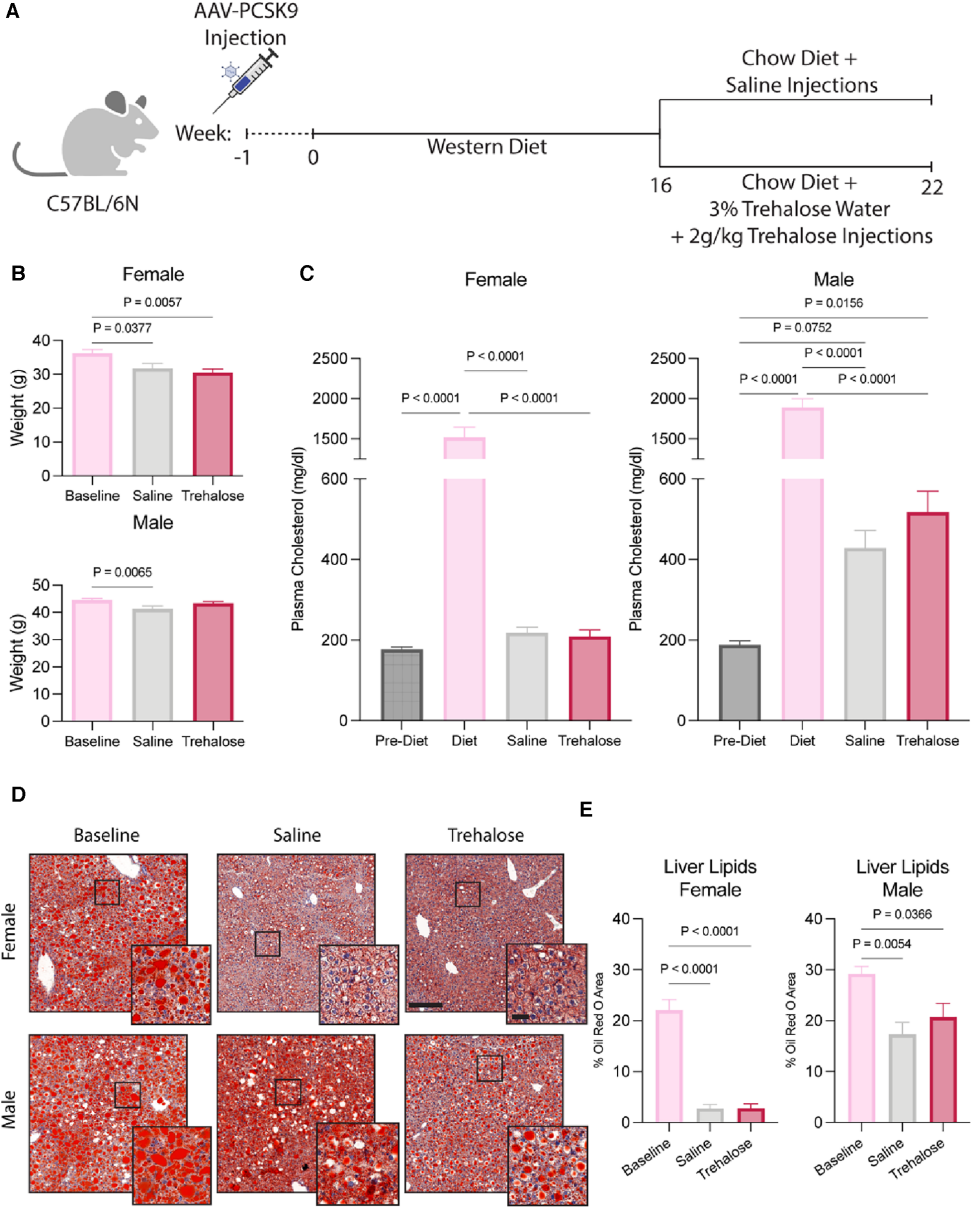
Trehalose does not alter body composition or circulating cholesterol. (**A**), Schematic of the experimental design. (**B**) Body weight of female and male mice during the 6 weeks of chow diet feeding (*n* = 10–11 males; *n* = 12 females). (**C**) Plasma cholesterol taken 1 week post AAV-PCSK9 injections, during Western Diet feeding and after 6 weeks of chow diet feeding (*n* = 10 males; *n* = 12 females). (**D,E**) Representative images and quantification of livers stained by Oil Red O (*n* = 4–5 males; *n* = 4–6 females). Scale bar = 200 µm and 50 µm in zoomed-in images. Statistical significance was assessed with a one-way ANOVA with a Holm-Sidak's *post hoc* test for multiple comparisons correction when comparing more than two groups.

Male and female C57BL/6N mice were injected with an adeno-associated virus (AAV) encoding gain-of-function in proprotein convertase subtilisin/kexin type 9 (PCSK9) to induce hypercholesterolemia. One week post-AAV injection, mice were fed a Western diet rich in fat and cholesterol to increase circulating cholesterol further and promote atherosclerotic plaque development. After 16 weeks of Western diet feeding, a randomized group of mice was sacrificed for baseline measurements, while the remaining mice were randomized for the regression treatment groups. The mice were switched to a chow (no added cholesterol and fat) diet for 6 weeks, as previously described (29), to initiate atherosclerosis regression. During regression, the mice received bi-weekly injections of trehalose or saline for 6 weeks. The mice in the trehalose treatment group also had water supplemented with 3% trehalose (Figure 1A).

The female regression groups exhibited weight loss during the 6 weeks of chow diet feeding. Male mice treated with trehalose did not lose weight, whereas the saline regression group exhibited weight loss (Figure 1B). Circulating plasma cholesterol at sacrifice also did not differ between saline and trehalose groups (Figure 1C). Interestingly, regressing male mice had higher levels of circulating plasma cholesterol (429 ± 43 mg/dl for saline and 518 ± 51 mg/dl for trehalose) as compared to females (218 ± 13 mg/dl for saline and 209 ± 16 mg/dl for trehalose) regardless of treatment groups (Figure 1C). Following the atherosclerosis regression paradigm, circulating plasma cholesterol returned to baseline following the diet switch from Western diet to chow in female but not male mice (Figure 1C). Unlike their female counterparts, post-regression plasma cholesterol levels of male mice were not restored to pre-Western diet levels and were twice that of pre-Western diet levels (Figure 1C).

During Western diet feeding, mice develop fatty livers, whereas upon atherosclerosis regression, hepatic steatosis resolves (29). Because fatty livers are associated with higher levels of circulating cholesterol (30) and we observed persistent elevated cholesterol concentrations in male mice even following the diet switch to chow (Figure 1C), we next assessed liver steatosis by Oil Red O staining and quantification. In female mice, as expected, Oil Red O staining was reduced following 6 weeks of chow diet feeding with no difference between the saline and trehalose groups (Figures 1D,E). Despite a significant reduction in hepatic lipid content in male mice following the diet switch to chow, liver steatosis was not fully resolved following atherosclerosis regression (Figures 1D,E). Although lipid droplet size decreased in regressing male mice, a significant amount of lipids remained in both saline and trehalose groups (Figures 1D,E). Overall, our data reveals a sexual dimorphism in whole-body lipid metabolism in mice undergoing atherosclerosis regression in the AAV-PCSK9 diet switch mouse model.

### Trehalose promotes plaque regression in female mice

**Figure 2:**
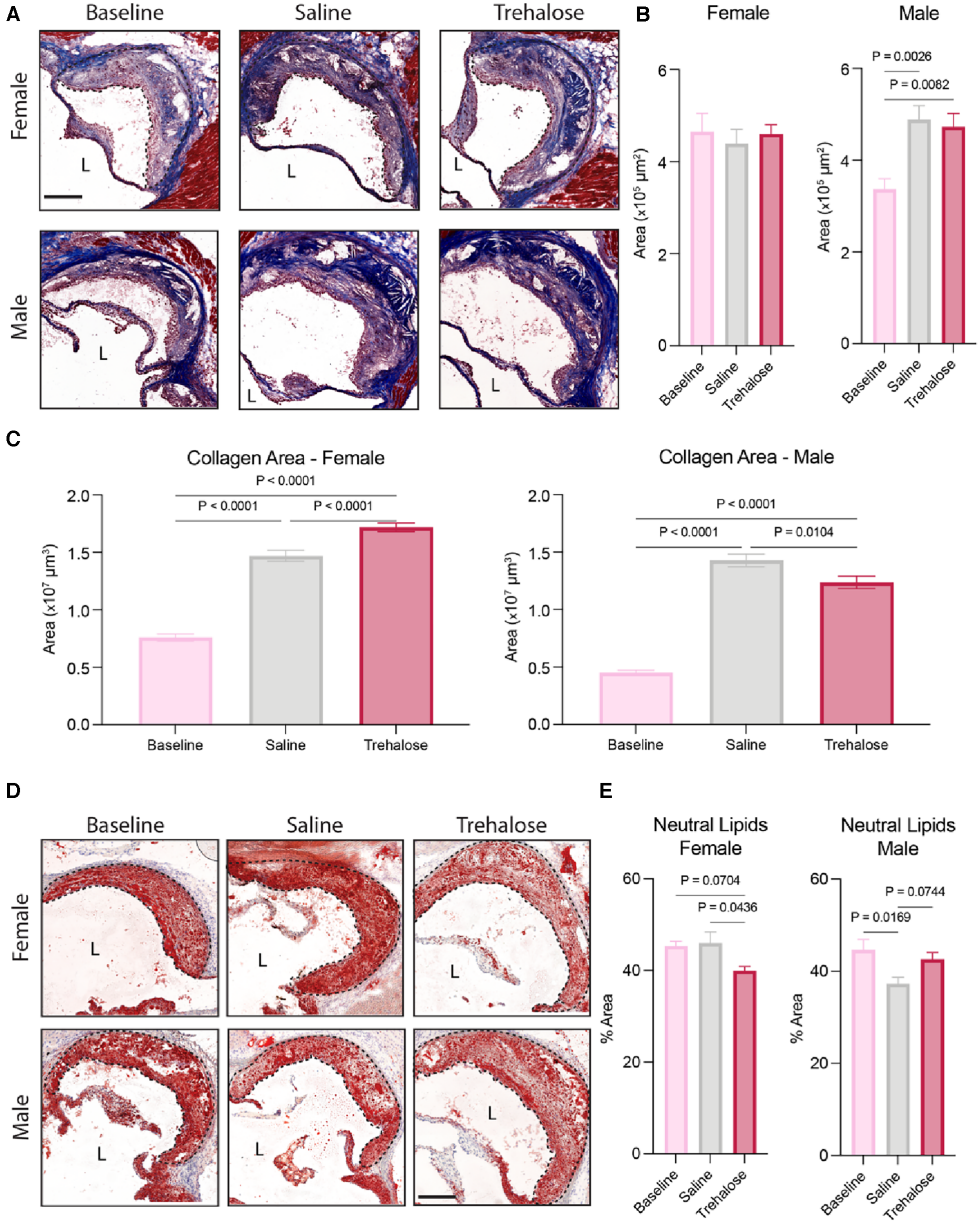
Trehalose promotes plaque stabilization in female mice. (**A**) Representative micrographs of aortic root stained with Masson Trichrome. (*n* = 8–11 males; *n* = 10–12 females). Scale bar = 200 µm. L = Lumen. (**B**) Quantification of atherosclerotic lesion area in the aortic root. (**C**) Quantification of the total collagen area throughout the aortic root (area under the curve) as quantified by the blue stain of Masson Trichrome. (**D**) Representative micrographs of aortic root stained with Oil Red O. Scale bar = 200 µm. L = Lumen. (**E**) Quantification of the percentage of the neutral lipid content of the total plaque area (*n* = 9–10 males; *n* = 10–12 females). Statistical significance was assessed using a one-way ANOVA with Holm-Sidak's *post hoc* test for multiple comparison correction when comparing more than two groups.

Regressing atherosclerotic plaques are characterized by one or more of the following: reduced size, reduced cholesterol content, dampened inflammation, reduced foam cell numbers, increased collagen content and/or increased fibrous cap size (5, 6). In female mice, atherosclerotic plaque growth was halted upon the reversal of hypercholesterolemia, as evidenced by comparable total plaque area in cross-sections of aortic roots in femal mice across the groups (Figures 2A,B). In male mice, plaque area increased significantly during atherosclerosis regression by 45% and 40% in the saline and trehalose groups, respectively (Figure 2B).

Given that collagen content is positively correlated with plaque stability during atherosclerosis regression (5), we next quantified plaque collagen content using Masson Trichrome staining. We observed increased collagen content in the mice fed a chow diet for 6 weeks regardless of treatment and sex (Figure 2C). In female mice, trehalose further increased plaque collagen content by 17% compared to saline, whereas in male mice, trehalose decreased collagen content by 13% compared to saline (Figure 2C).

Finally, to quantify plaque neutral lipids, we stained aortic roots with Oil Red O (Figure 2D). In female mice, trehalose treatment significantly reduced lipid content as compared to saline-treated mice (Figure 2E). The chow diet reduced lipid content in the male mice treated with saline but not trehalose (Figure 2E). Collectively, these data demonstrate that plaque growth is halted upon the reversal of hypercholesterolemia in female mice as compared to male mice and that trehalose reduces the neutral lipid content while increasing collagen deposition in female but not male mice.

### Differential regulation of autophagy in male and female mice

**Figure 3:**
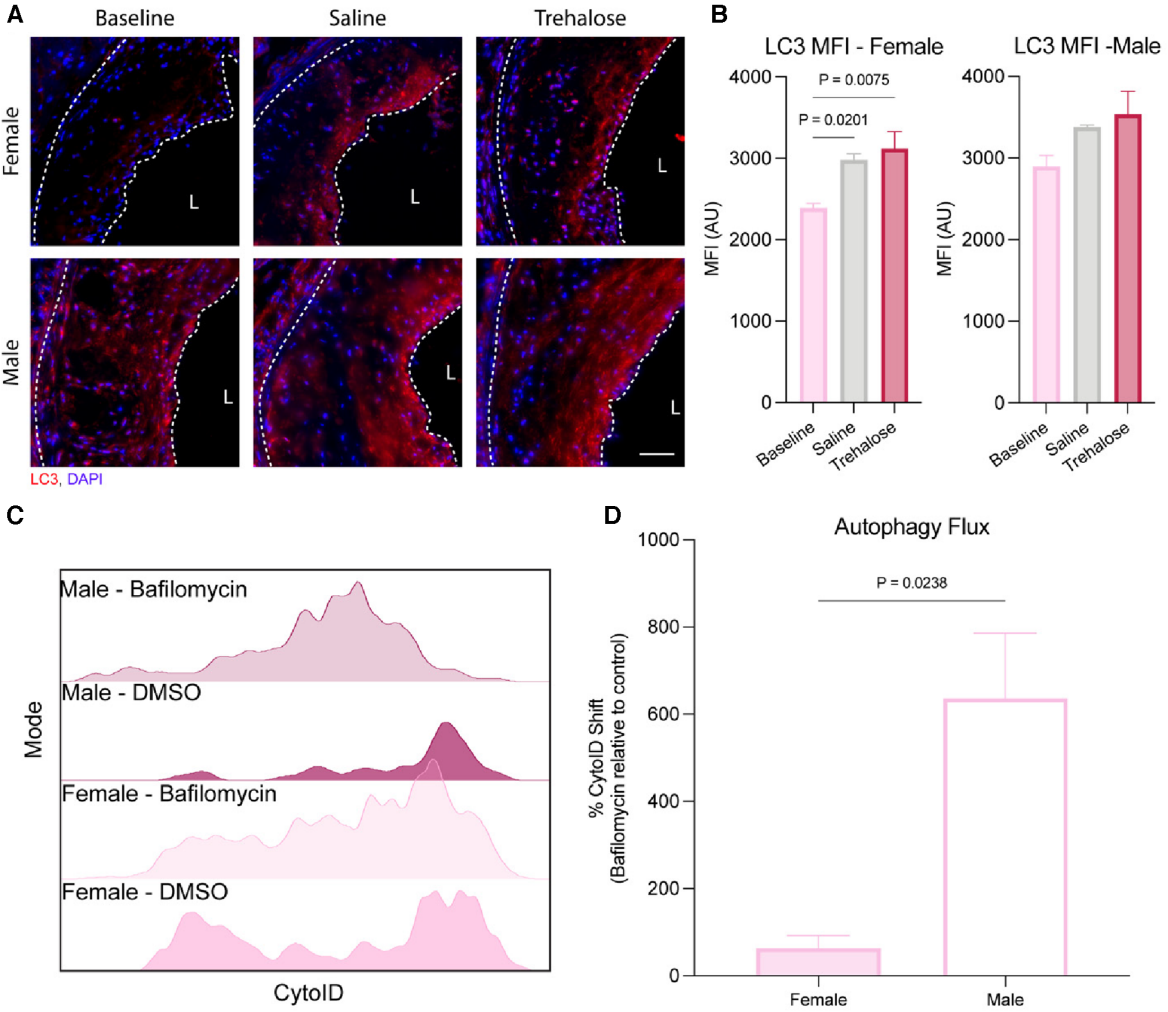
Autophagy is differentially regulated between female and male mice. (**A**) Representative immunofluorescence of the aortic root stained with the autophagosome marker LC3 (red) and nucleus (blue). Scale bar = 50 µm. (**B**) Quantification of the mean fluorescence intensity (MFI) of the autophagosome marker LC3 within the total plaque area (*n* = 6 males and females). (**C**) Representative histogram of CytoID fluorescence intensity (*n* = 3 males and females). (**D**) Fold change of the CytoID MFI to Bafilomycin (*n* = 3 males and females). Statistical significance was assessed with an unpaired *t*-test when comparing two groups or one-way ANOVA with Holm-Sidak's *post hoc* test for multiple comparisons correction when comparing more than two groups.

To determine if atherosclerosis regression and/or trehalose activate autophagy, we performed immunostaining of aortic roots with the autophagosome marker microtubule-associated protein 1A/1B-light chain 3 (LC3). LC3 is a crucial component of autophagy that gets incorporated into the membranes of autophagosomes during their biogenesis and, as such, is a specific marker of autophagy (31). Atherosclerosis regression did not increase LC3 expression in the plaques of male mice, while we observed increased LC3 expression in those of female mice treated with either saline (25%) or trehalose (30%) (Figures 3A,B).

Autophagy is a dynamic process best quantified as the flux of autophagosomes to lysosomes (32). During autophagy, the cargo to be degraded is engulfed by autophagosomes that fuse with nearby lysosomes. However, in the presence of bafilomycin that inhibits lysosomal acidification, lysosome function is inhibited, leading to a buildup of autophagosomes in bafilomycin-treated cells. Therefore, the accumulation of autophagosomes upon bafilomycin treatment reflects a high cellular autophagy flux. Aortic arches were digested and treated with bafilomycin *ex vivo* to quantify autophagy flux during atherosclerosis regression, as we previously did for atherosclerosis progression studies (19). Aortic digests were stained with the autophagosome dye CytoID, which labels autophagic compartments. In parallel, lipid-rich (Lipid^hi^) arterial foam cells were identified using the lipid dye BODIPY, with leukocyte-derived foam cells identified as CD45^+^ and MHC-II^+^ (19).

Using flow cytometry of aortic digests, we observed that Lipid^hi^CD45^+^MHC-II^+^ leukocyte foam cells from male aortas had significantly higher autophagy flux at baseline than the females (Figures 3C,D). Because CytoID accumulates in lysosomes upon autophagy inhibition by bafilomycin treatment, the dye is quenched, decreasing the fluorescence intensity. As seen in Figure 3C, the reduction in CytoID fluorescence upon bafilomycin treatment was most striking in Lipid^hi^CD45^+^MHC-II^+^ leukocyte foam cells from male as compared to female mice, which was quantified in Figure 3D. These results highlight sex differences in basal autophagy flux, whereby macrophage foam cells from female plaques have a markedly lower basal autophagy flux than males. Interestingly, the autophagy flux was similar *in vitro* in mouse peritoneal macrophages isolated from male compared to female mice in both unloaded and lipid-loaded conditions (Supplementary Figure S2), suggesting that the sex differences observed in plaque macrophage autophagy flux are not attributable to cell-intrinsic sex differences, but rather to more complex physiological sex differences between male and female atherosclerotic mice.

### Trehalose reduces inflammation in female mice

**Figure 4:**
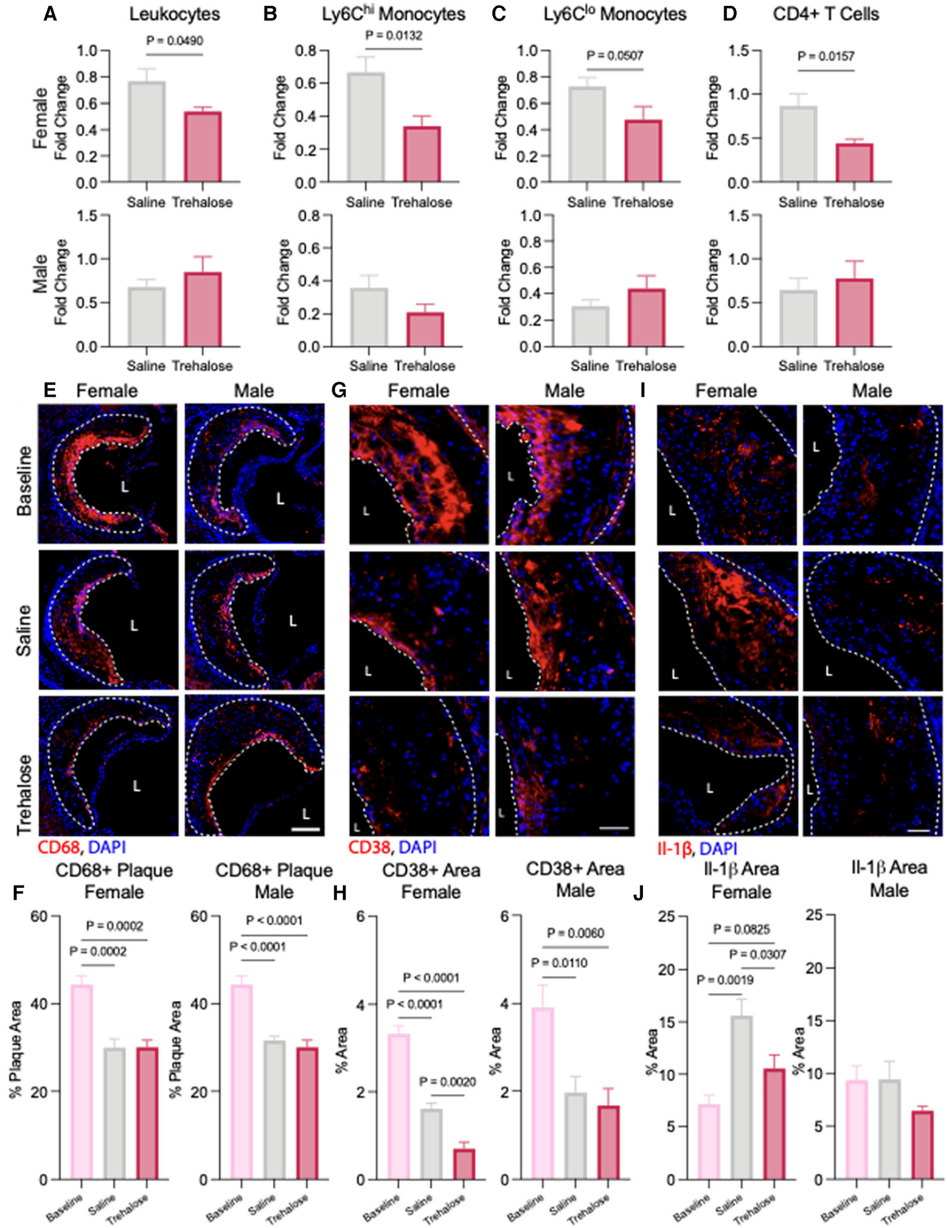
Trehalose reduces inflammation in female mice. (**A**) Fold change of the total circulating (**A**) leukocytes, (**B**) Ly6C^hi^ monocytes, (**C**) Ly6C^lo^ monocytes and (**D**) CD4+ *T* cells following 5 weeks of chow diet and treatment over the baseline total cells. (**E**) Representative immunohistochemistry images of the aortic roots stained with CD68 (red) and nucleus (blue). Scale bar = 50 µm. L = Lumen (**D**) Quantification of the percentage of CD68 positive area of the total plaque (*n* = 6 males and females). (**G**) Representative immunohistochemistry of aortic roots stained with inflammatory marker CD38 (red) and nucleus (blue). Scale bar = 50 µm. (**H**) Quantification of the percentage of CD38 positive and CD68 positive area found in the total plaque area (*n* = 6 males and females). (**I**) Representative immunohistochemistry of aortic roots stained with inflammatory marker Il-1β (red) and nucleus (blue). Scale bar = 50 µm. (**J**) Quantification of the percentage of Il-1β positive area found in the total plaque area (*n* = 6 males and females). Statistical significance was assessed with an unpaired *t*-test when comparing two groups or one-way ANOVA with Holm-Sidak's *post hoc* test for multiple comparisons correction when comparing more than two groups.

Quantification of circulating immune cells at baseline and 5 weeks of atherosclerosis regression revealed a significant reduction in the total number of leukocytes after chow diet feeding in the male saline and trehalose groups and in the female trehalose group (Supplementary Figure S1A). However, during atherosclerosis regression in female mice, trehalose only significantly reduced circulating immune cells and leukocytes (Figure 4A). Monocytes can be further subcategorized into two different subsets: Ly6C^hi^ and Ly6C^lo^. Ly6C^hi^ monocytes are elevated in hypercholesterolemia and are the major subtype recruited to atherosclerotic plaques (33). In our study, female mice treated with trehalose had significantly fewer Ly6C^hi^ monocytes, with Ly6C^lo^ monocytes trending toward a decrease (Figures 4B,C). In addition to monocytes, T cells, particularly CD4^+^ T cells, were found to be pro-atherogenic (34). Interestingly, CD4^+^ T cells were also significantly reduced in trehalose-treated female mice compared to saline controls (Figure 4D), while CD8+ T cells were unchanged (Supplementary Figure S1B). Although total leukocyte numbers were reduced in male mice upon atherosclerosis regression (Supplementary Figure S1A), leukocytes, monocytes and T numbers were unchanged in the trehalose group compared to the saline group (Figures 4A–D).

To quantify the macrophage content of atherosclerotic plaques, we performed immunofluorescence staining of the aortic root with the macrophage marker CD68. In line with reduced levels of circulating Ly6C^hi^ monocytes observed in the regressing mice (Figure 4B), we observed a significant decrease in plaque macrophage content in both male and female mice during atherosclerosis regression in saline and trehalose groups (Figures 4E,F). To evaluate the inflammatory status of plaque macrophages, we performed immunostaining of aortic roots with the pro-inflammatory macrophage marker CD68 and the pro-inflammatory marker CD38. The latter is described as a cell surface marker expressed by pro-inflammatory macrophages (35). In both male and female mice, atherosclerosis regression markedly reduced CD68^+^CD38^+^ plaque area in the saline and trehalose groups (Figures 4G,H). Trehalose treatment further reduced CD68^+^CD38^+^ plaque area in female mice but not male mice (Figure 4H), indicating increased inflammation resolution in trehalose-treated female mice.

Finally, because macrophage autophagy promotes the clearance of the NLRP3 inflammasome to limit the production of pro-inflammatory interleukin (IL)-1β in atherosclerosis (13), we next sought to quantify plaque IL-1β levels in our study groups. Surprisingly, IL-1β levels were upregulated by 32% in saline-treated female mice relative to baseline, while trehalose treatment mitigated this increase back to baseline levels (Figures 4I,J). IL-1β levels were not significantly different between the groups in male mice (Figures 4I,J). Overall, trehalose treatment in female mice reduced circulating inflammatory immune cells, plaque inflammatory macrophages and plaque IL-1β.

## Discussion

Even with the development of cholesterol-lowering drugs, cardiovascular diseases significantly burden the healthcare system (36). Indeed, cholesterol-lowering drugs may slow disease progression and result in mild plaque shrinkage (37), but they have yet to promote the complete reversal of atherosclerosis. Rapamycin and its derivatives (rapalogs) have been used in drug-eluting stents to promote autophagy via mTORC1 inactivation. These studies have produced small but significant reductions in atherosclerosis (38–40), suggesting that plaque autophagy activation is an exciting target to promote atherosclerosis regression. However, the systemic inhibition of mTORC1 by rapamycin and rapalogs has detrimental side effects, including immunosuppression, hyperglycemia, insulin resistance and dyslipidemia (41), thereby making it unsuitable for the reversal of atherosclerosis.

In recent years, TFEB activation using the disaccharide trehalose has gained attention as a potential therapeutic approach to promote autophagy. By causing low-grade lysosomal stress to activate TFEB, trehalose drives the transcription of genes involved in lysosome and autophagosome biogenesis, thereby increasing autophagy flux (23, 24). In pre-clinical models of cardiovascular disease, trehalose reduces disease burden (26, 42, 43). Moreover, recent clinical trials have demonstrated that trehalose is safe for use and improves arterial dilation in middle and old-aged adults (44, 45). Trehalose is currently under investigation for the treatment of amyotrophic lateral sclerosis (46), Parkinson's disease (47) and spinocerebellar ataxia (48). Excitingly, TFEB activation by trehalose occurs in a mTORC1-independent manner (49, 50), making the use of trehalose to reverse atherosclerosis a promising therapeutic approach.

Here, we tested whether trehalose could ameliorate atherosclerosis in the AAV-PCSK9 diet switch mouse model. This model employs overexpression of a gain-of-function PCSK9 mutant in C57BL/6N mice and 16 weeks of Western diet feeding to establish atherosclerosis (baseline). Then, atherosclerosis regression was initiated by a diet switch to chow, and the mice were treated with trehalose or saline for 6 weeks. As previously demonstrated (43, 51), the administration of trehalose did not alter body composition or plasma cholesterol levels in mice in our study. Circulating plasma cholesterol returned to baseline following the diet switch to chow in female mice. In contrast, the circulating plasma cholesterol of male mice was not restored to baseline but was lowered to double that of pre-Western diet levels. In female mice undergoing atherosclerosis regression, we observed positive remodelling of atherosclerotic plaques, including the halting of plaque growth, reduced plaque neutral lipid content and inflammatory macrophages concomitant with increased plaque collagen and autophagosomes. Moreover, trehalose futher enhanced the positive remodeling of atherosrotic plaques in regressing female mice, futher reducing plaque neutral lipid and inflammatory macrophages and potentiating plaque collagen deposition and autophagy as compared to controls. In contrast, plaque growth was not halted in male mice undergoing atherosclerosis regression. Rather, we observed increased plaque area in regressing male mice, potentially due to a moderate hypercholesterolemia that persisted following the switch to chow diet in both trehalose- and saline-treated groups.

In addition to reductions in plasma cholesterol, the mitigation of inflammation is critical to atherosclerosis resolution (8). Upon hypercholesterolemia, the production of immune cells is dysregulated and skewed towards myelopoiesis, leading to an increase in the circulating monocyte pool (52). This increase in circulating monocytes perpetuates monocyte recruitment to the arterial intimal space, promoting atherosclerosis progression. Conversely, in atherosclerosis regression, the recruitment of circulating monocytes is reduced, resulting in reduced plaque macrophage numbers (53). In conjunction with enhancing autophagy, several studies show that TFEB activation alleviates inflammation (54–56). Also, the activation of TFEB attenuates endothelial inflammation by decreasing the expression of adhesion molecules and chemokines, thereby reducing monocyte adhesion and infiltration (55, 57). In our study, we observed a reduction in the total number of circulating leukocytes after chow diet feeding in the saline and trehalose regression groups compared to baseline in both sexes. Meanwhile, trehalose only further reduced circulating leukocytes relative to the saline group in females. Moreover, trehalose further reduced pro-inflammatory Ly6C^hi^ monocytes and CD4^+^ T cells relative to saline in female mice, indicative of increased inflammation resolution in trehalose-treated female mice.

In addition to employing circulating immune cell phenotyping to assess inflammation in atherosclerosis, levels of the pro-inflammatory cytokine IL-1β can be used as a local marker of inflammation. Plaque IL-1β production is highest in patients with more complex, less stable plaques (58) and conversely, the Canakinumab Antiinflammatory Thrombosis Outcome Study (CANTOS) demonstrated that resolution of residual inflammation using a monoclonal antibody targeting IL-1β antibody reduces major adverse cardiovascular events (59). Interestingly, in our study, we observed that a switch to a chow diet significantly increased IL-1β levels in plaques of the female mice as compared to baseline. While this observation seems counterintuitive because inflammation resolution is critical to atherosclerosis regression (8), IL-1β is required for the maintenance of a fibrous cap rich in smooth muscle cells and collagen (60). In regressing female mice, trehalose mitigated the IL-1β increase back to baseline levels, while plaque IL-1β levels were not significantly different between the groups in male mice, highlighting sex differences in inflammation resolution during atherosclerosis regression. Further studies are needed to understand the mechanisms by which IL-1β levels are altered in the different mouse cohorts, and whether this is occuring via autophagy- and/or inflammasome-dependent mechanisms.

Interestingly, autophagy flux comparisons revealed lower basal autophagy in the plaques of female mice than in male mice. This is consistent with previous observations demonstrating that male mice have a higher autophagy flux (61, 62) and require higher doses of rapamycin than female mice to activate autophagy (63). This elevated autophagic flux in males can be beneficial during Alzheimer's disease by preventing the accumulation of cytotoxic amyloid beta plaques (61) or detrimental in the case of myocardial infarction, where excessive autophagy promotes cardiomyocyte death (56). Here, the higher autophagy flux observed in male mice may have had a protective effect by reducing atherogenesis, as observed by the smaller plaque sizes at baseline in males compared to females. The heightened ability of females to undergo atherosclerosis regression may lie in their inherent higher capacity to perform cholesterol efflux (64) given higher circulating HDL levels in female mice (65), as well as increased hepatic lipolysis in female mice relative to males (66, 67). Lastly, treatment with statins leads to more significant regression in females than males (68), highlighting the differential capacity of plaque regression between sexes. To conclude, our study demonstrates key disparities in autophagy flux between sexes, leading to significant differences in atherosclerosis resolution. Elucidating the regulation of autophagy in both sexes is critical to harnessing autophagy activation to promote plaque regression as a therapeutic approach for the treatment of atherosclerosis.

## Data Availability

The original contributions presented in the study are included in the article/Supplementary Material, further inquiries can be directed to the corresponding author.

## Supplementary material

Supplementary Figure 2 Autophagy flux is equivalent in mouse macrophages isolated from male as compared to female mice. (**A**) Immunoblotting of peritoneal macrophages isolated from male or female mice and cultured for 2h in the presence or absence of Bafilomycin (*n* = 4). (**B**) Immunoblotting of male or female mouse peritoneal macrophages lipid-loaded with 50 μg/mL of aggregated LDLs for 24h before treatment with or without Bafilomycin (*n* = 4).
