# Supplementary material for: Trehalose promotes atherosclerosis regression in female mice
Source: Front Cardiovasc Med. 2024 Feb 16;11:1298014. doi: 10.3389/fcvm.2024.1298014 (PMC10906268; doi:10.3389/fcvm.2024.1298014)
Supplement: Supplementary file 1 [file Table1.docx]

***Supplementary Material***

**1. Supplementary Data**

**
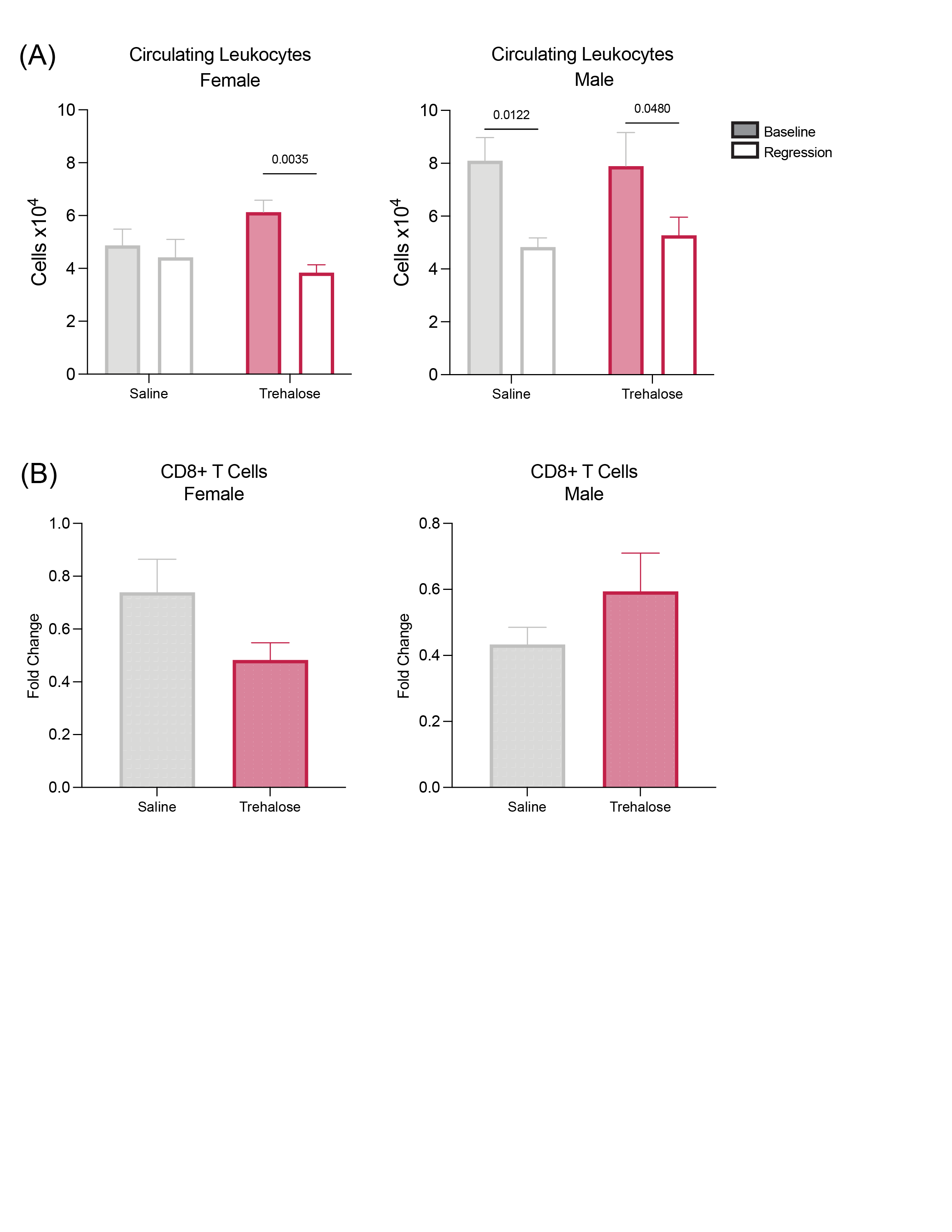
Supplementary Figure 1.** (A) Circulating leukocytes numbers taken at baseline (16 weeks of diet) and 5 weeks of chow diet feeding. (B) Fold change of the total circulating CD8+ T cells following five weeks of chow diet and treatment over the baseline total cells. . Statistical significance was assessed with an unpaired t-test when comparing two groups or two-way ANOVA when comparing more than two groups.

**2. Supplementary Table**

**Supplementary Table 1: List of antibodies**

| Target Antigen | Catalog # | Supplier |
| --- | --- | --- |
| Ly6C-BV610 | 563011 | BD Bioscience |
| CD8A-BV510 | 563068 | BD Bioscience |
| CD11b-BV421 | 562605 | BD Bioscience |
| CD4-PerCP-Cy5.5 | 561115 | BD Bioscience |
| CD45-APC-Cy7 | 561037 | BD Bioscience |
| CD3e-APC | 553066 | BD Bioscience |
| Purified Rat Anti-Mouse CD16/CD32 | 553141 | BD Bioscience |
| CD45-APC-Fire 750 | 147714 | Biolegend |
| I-A/I-E-BF785 | 107645 | Biolegend |
| LC3 | PM036 | MBL International |
| CD45-AF647 | 103124 | Biolegend |
| CD68-AF750 | NBP2-33337AF750 | Novus |
| Il-1β | ab254360 | Abcam |
| CD38 | ab61400 | Abcam |
| Goat anti-rabbit Alexa Fluor 555+ | A32732 | Invitrogen |
| Donkey anti-rabbit Alexa Fluor 555+ | A32794 | Invitrogen |
| Donkey anti-rat Alexa Fluor 488+ | A48269 | Invitrogen |
| Alexa Fluor® 647 Rat IgG2a, κ Isotype Ctrl Antibody | 400526 | Biolegend |
| Rat IgG2a Isotype Control (54447) [Alexa Fluor® 750] | IC006S | Novus |
