# Supplementary figures and images for: Trehalose promotes atherosclerosis regression in female mice
Source: Front Cardiovasc Med. 2024 Feb 16;11:1298014. doi: 10.3389/fcvm.2024.1298014 (PMC10906268; doi:10.3389/fcvm.2024.1298014)

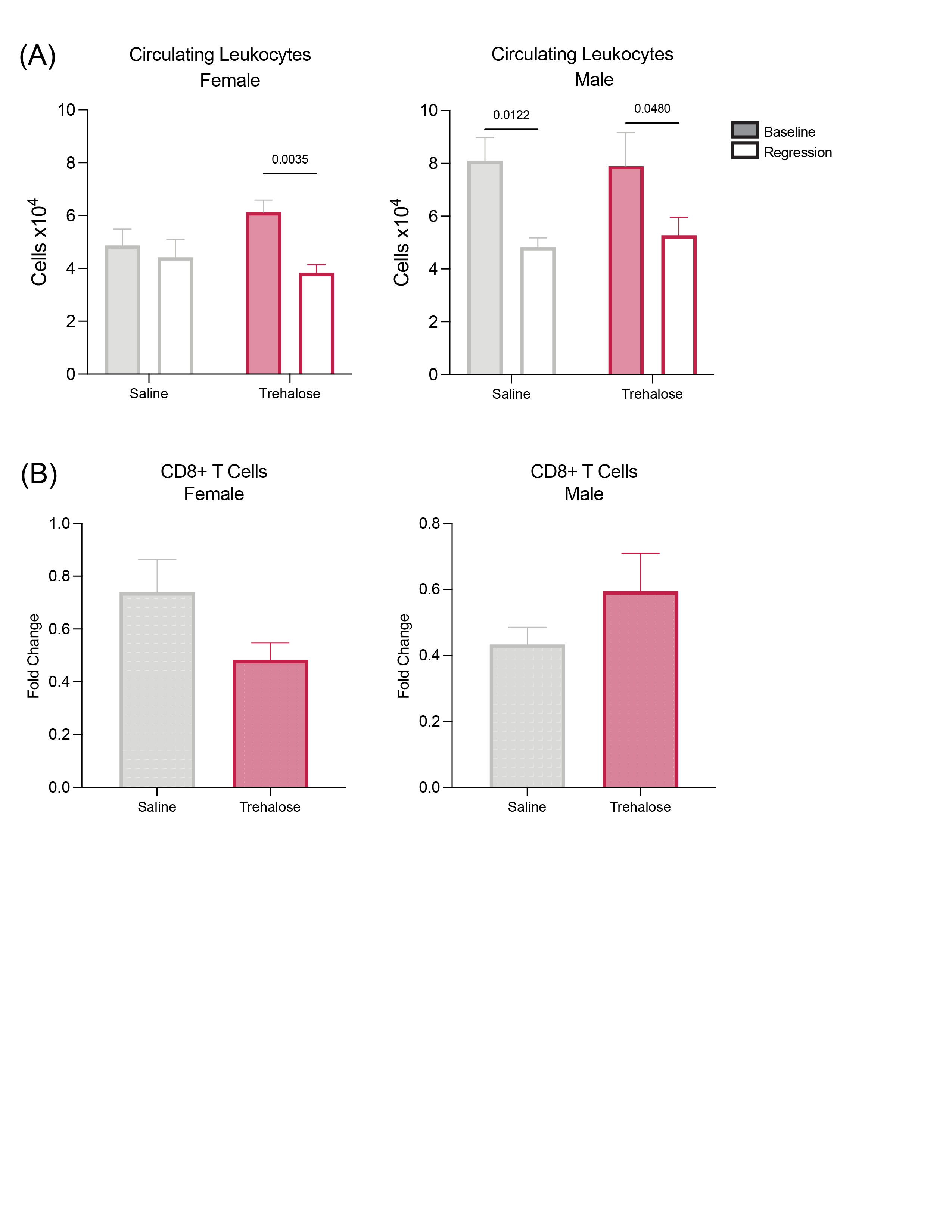

Supplement: Supplementary file 2 [file Image1.png]

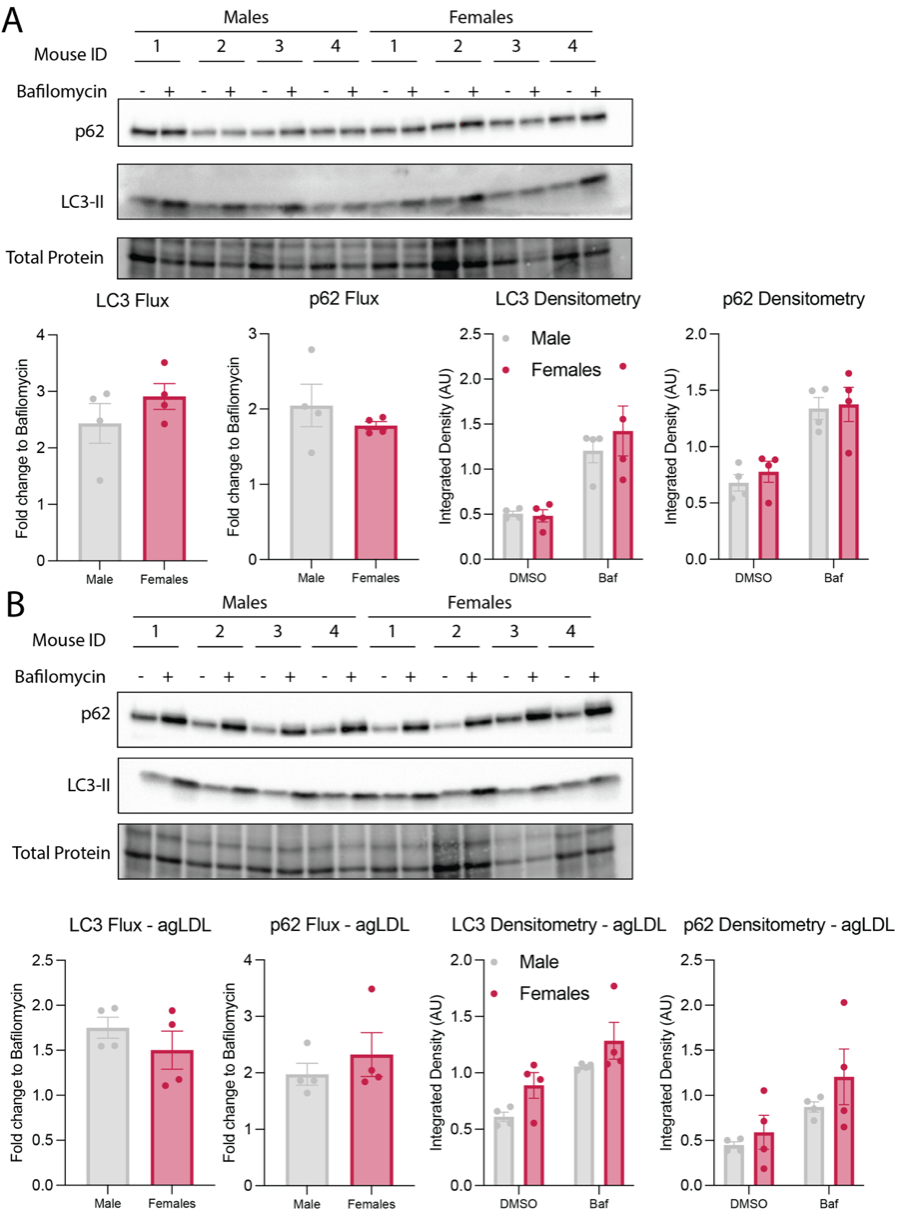

Supplement: Supplementary Figure 2 — Autophagy flux is equivalent in mouse macrophages isolated from male as compared to female mice. (A) Immunoblotting of peritoneal macrophages isolated from male or female mice and cultured for 2h in the presence or absence of Bafilomycin (n = 4). (B) Immunoblotting of male or female mouse peritoneal macrophages lipid-loaded with 50 μg/mL of aggregated LDLs for 24h before treatment with or without Bafilomycin (n = 4). [file Image2.png]
